# How to Establish the Bipolar Forceps Dissection Method in Robotic Inguinal Hernia Repair

**DOI:** 10.1002/ags3.12535

**Published:** 2021-12-14

**Authors:** Takuya Saito, Yasuyuki Fukami, Shunichiro Komatsu, Kenitiro Kaneko, Tsuyoshi Sano

**Affiliations:** ^1^ Division of Gastroenterological Surgery Department of Surgery Aichi Medical University Nagakute Japan

**Keywords:** bipolar forceps, inguinal hernia, laparoscopy, learning curve, robotic surgical procedures

## Abstract

The number of robotic inguinal hernia repair (RIHR) surgeries performed by younger surgeons and surgical residents has been growing worldwide. As a result, there has been growing interest in the pace at which surgeons develop their competencies. In Japan, the number of robotic surgeries with the double bipolar technique for gastric cancer is increasing. We devised an RIHR technique for a right‐hand‐dominant surgeon. This article describes the procedure and step‐by‐step instructions for this technique. We also assessed the learning curve of a surgeon experienced in the laparoscopic transabdominal preperitoneal (TAPP) approach and robotic gastrectomy. This was a retrospective review of 31 inguinal hernia patients (40 lesions) between December 2018 and April 2021 operated by a single surgeon. The cumulative summation technique (CUSUM) was used to construct a learning curve for robotic proficiency by analyzing the times for peritoneal flap creation, mesh placement, and peritoneal closure. The postoperative course, namely, the length of hospital stay, 30‐d complications, and 30‐d readmission rates, was evaluated. The CUSUM graph for the total time for each phase indicated an initial decrease at lesion 12 and another decrease at lesion 36, generating three distinct performance phases: learning (n = 12 procedures), competence (n = 24), and mastery (n = 4). Between the early and late periods, no significant differences in patient characteristics or surgical outcomes were found. The learning curve for this technique was divided into three performance phases, and the technique was safely achievable in 36 procedures by a surgeon with previous experience in laparoscopic TAPP.

## Graphical abstract

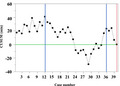

## 1 INTRODUCTION

Recently, the adoption of robotic surgery for inguinal hernia repair has increased exponentially, mainly in the USA.^1^ The international guidelines for inguinal hernia management report that 100 supervised laparo‐endoscopic repairs are needed to achieve the same results as open mesh surgery techniques, such as the Lichtenstein procedure.^2^ However, there are few reports evaluating the learning curve (LC) of robotic inguinal hernia repair (RIHR), and there is no consensus on the criteria for operating surgeons. In comparison, in Japan robotic surgery is increasingly being performed for gastric cancer and rectal cancer.^3^, ^4^ Many surgeons perform robotic gastrectomy (RG) using the double bipolar technique, and a decrease in postoperative pancreatic fistula formation in radical lymph node dissection has been reported.^5^, ^6^ Uyama et al reported that Maryland forceps, which are controlled by the surgeon's dominant hand, are appropriate for precise dissection because of their articulation, tapered tip, ability to hold the tissues under dissection, and efficient hemostasis.^7^ Thus, we established our surgical technique by introducing RIHR with the transabdominal preperitoneal approach (R‐TAPP) using the bipolar method. In this article, we describe the surgical technique of this method, focusing on the LC of a single surgeon. We used the cumulative summation technique (CUSUM), which is a popular and reliable method for evaluating the LC of a surgical procedure.

## 2 PATIENTS AND METHODS

Between December 2018 and April 2021, consecutive patients who underwent R‐TAPP using the bipolar forceps dissection technique at our institution were investigated. All operations were performed under a protocol designed at our hospital by a single qualified surgeon (Takuya Saito) who completed the LC for laparoscopic inguinal hernia repair with the transabdominal preperitoneal approach (L‐TAPP) prior to performing R‐TAPP.^8^, ^9^ In addition, the operating surgeon performed more than 50 RGs within the study period. The patients' demographics, clinical characteristics, intraoperative data (console time, total operative time, and blood loss), and 30‐day postoperative outcomes (overall complications, length of stay, and readmission) were reviewed. Postoperative complications comprised surgical site infection, urinary retention, small bowel obstruction, ileus, continuous severe pain, and abdominal abscess.

All patients provided written informed consent before undergoing surgery. In December 2018, we introduced R‐TAPP as a treatment option after obtaining approval from the Ethical Committee of Aichi Medical University (AMU) Hospital. This study was approved by the Institutional Review Board of our institution (No. 2019‐086) and was performed in accordance with the 1964 Declaration of Helsinki and its later amendments or comparable ethical standards.

### 2.1 Access, port position, and instruments

All procedures were performed using the da Vinci Xi robotic platform (Intuitive Surgical, Sunnyvale, CA). Patients were placed in the Trendelenburg position, and a supraumbilical camera trocar was placed in the midline. Two 8‐mm working trocars were placed at least 3 cm from the costal arch and anterior superior iliac spine on each side. The robot was docked from the left or right, according to the side of the hernia. We used Cadiere forceps with the left hand and Maryland bipolar forceps (Intuitive Surgical) with the right hand. The Maryland bipolar forceps were connected to a VIO 300D electrosurgical generator (Erbe USA, Marietta, GA) in the forced coagulation mode.^8^, ^10^

### 2.2 Surgical techniques and step‐by‐step technique

#### 2.2.1 Peritoneal flap creation

**FIGURE 1:**
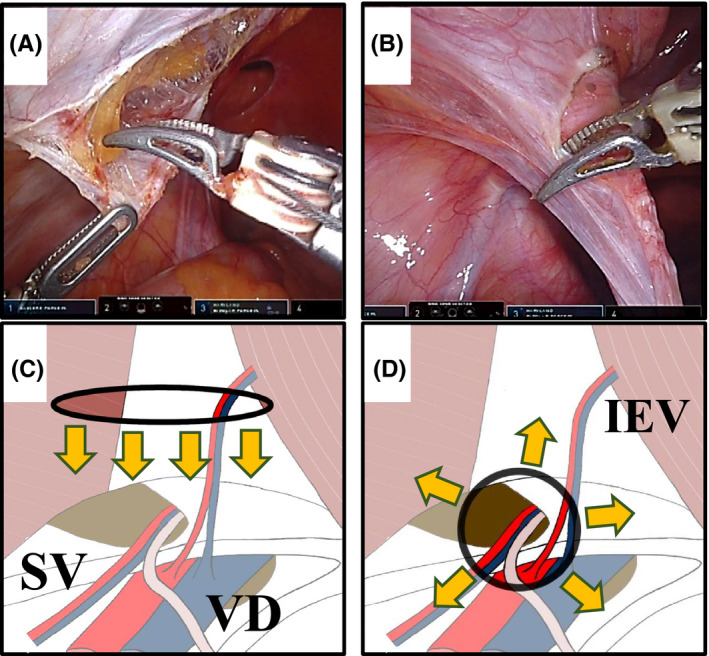
The peritoneal incisional point. (A/C) The first step in robotic TAPP hernia repair is to start the peritoneal incision at the upper 3–4 cm of the hernia defect. We show the picture in (A) and the schema in (C). The black line represents the peritoneal incision and the yellow arrows represent the direction of the dissection. SV, spermatic vessels; VD, vas deferens. (B/D) The first step in robotic TAPP repair for indirect hernias is to reverse the hernia sac into the abdominal cavity. Next, an annular incision is made in the hernia sac. We show the picture in (B) and the schema in (D). The black line represents the circular peritoneal incision and the yellow arrows represent the direction of the dissection. IEV, inferior epigastric vessels

**FIGURE 2:**
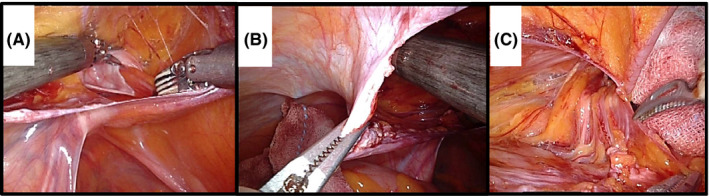
Peritoneal flap creation using forceps. (A) Traction and counter‐retraction are shown for separating the transversalis fascia and hernia sac in an indirect hernia. (B) The tip of the Maryland forceps is inserted and dissection proceeds. (C) The transversalis fascia is dissected for parietalization of the spermatic cord components

**FIGURE 3:**
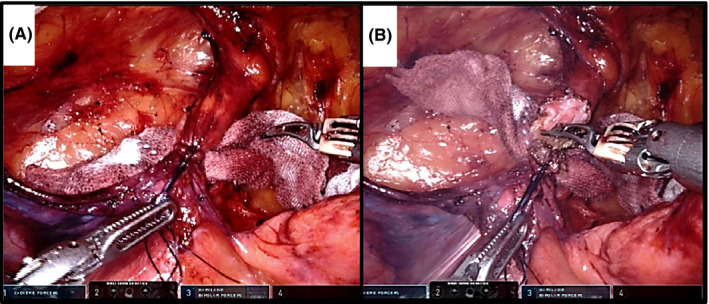
(A) Parietalization of the cord components can be safely performed in direct hernia repair. (B) The hernia sac is separated from the cord structures, ligated, and dissected

We started the peritoneal incision at the upper 3‐4 cm of the hernia defect (Figure 1A,C), 1 cm above the level of the anterior superior iliac spine (ASIS). This was an ideal fit for placing a 10‐cm wide mesh. The peritoneum was pulled away (down and medial) by the left and right forceps (this procedure is demonstrated in Video S1). Carbon dioxide was used to fill the pre‐transversalis space to facilitate plane separation. The flap cut was performed horizontally, lateral to medial, while performing secure hemostasis with the Maryland forceps. Figure 1B,D shows the standardized surgical technique for L‐TAPP that was used in the present study and the prior incision of the hernia sac (the procedure is demonstrated in Video S2). We modified the technique to create a peritoneal flap from the cranial position of the hernia defect once the robotic surgical technique was familiarized. This approach is difficult to close the peritoneal flap with the straight instruments in L‐TAPP. However, due to the amplification of the surgeon's laparoscopic technique by the various advanced functions in DVSS (high‐resolution 3D images, tremor filtering, the EndoWrist function), suture closure of the peritoneal flap can be performed with no difficulty. In addition, because the peritoneal flap is not hanging down, the mesh can be easily fixed with sutures. A peritoneal flap was dissected between the peritoneum and transversalis fascia, identifying the inferior epigastric vessels (IEV) as landmarks. Medially, dissection was extended to Retzius space, 1–2 cm below the pubic tubercle, identifying Cooper's ligament (CL) in the process. Dissection was performed until the white line of the rectus abdominis was crossed, while carefully preserving the vesicohypogastric fascia to avoid bladder injury and postoperative urinary retention. Usually, this space contains loose areolar tissue, which allows for easy blunt dissection with two‐handed forceps. In direct hernia repair, this method was also effective in traction and counter‐retraction to separate the transversalis fascia and hernia sac (Figure 2A). Laterally in the IEV and spermatic vessels (SV), to prevent lateral femoral cutaneous nerve injury and blood oozing, the robotic surgical technique of inserting the tip of the Maryland forceps was effective in maintaining the dissection before preperitoneal fatty tissue (Figure 2B). The lateral border of this dissection is the ASIS. The meticulous procedure by Maryland forceps of R‐TAPP was most effective for parietalization of spermatic cord components (Figure 2C). The peritoneum of the vas deferens and spermatic vessels could be safely removed by maintaining proper inferior traction, precise dissection, and hemostatic manipulation (Figure 3A). Spermatic cord lipomas were easily resected, if detected. The hernia sac was separated from the cord structures, ligated, and dissected (Figure 3B).

#### 2.2.2 Mesh placement and fixation

**FIGURE 4:**
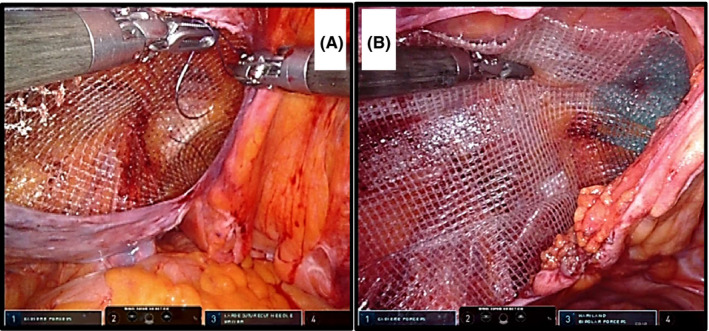
Mesh placement using Maryland forceps. (A) Polypropylene mesh (15 × 10 cm size) is used for suturing and fixation. (B) Initially, the Parietex ProGrip (Self‐Fixating Mesh, Medtronic) is placed and fixed using Maryland forceps

We used a polypropylene mesh (15 x 10 cm) that was sutured and fixed to the left and right sides of the IEV, CL, and the rectus muscle (Figure 4A). The mesh was rolled up and inserted through the working trocar. In the process of establishing our surgical technique, the polypropylene mesh was fixed with tacks by the bedside surgeon or a self‐fixating mesh (Parietex ProGrip; Medtronic, Dublin, Ireland; Figure 4B) was used (the procedure is demonstrated in Video S3). The self‐fixating mesh is difficult to insert from the 8 mm trocar. Therefore, when we envision the use of this mesh, a 12 mm‐umbilical trocar is placed from the beginning of the operation.

#### 2.2.3 Peritoneal flap closure

The intraperitoneal pressure was decreased to 8 mmHg to reduce tension during suturing. We started suturing from medial to lateral using 3‐0 Stratafix (1/2 circle needle; Ethicon, Raritan, NJ), and closed the opened peritoneum with barbed sutures. To adjust the alignment of the anterior and posterior flaps, we sutured halfway around for both flaps.

### 2.3 Statistical analysis

Continuous variables were expressed as median (range) and were compared between the early and late periods using the chi square test, the Mann–Whitney *U* test, or Fisher's exact probability test, as appropriate. All *P* values were two‐sided, and *P* < .05 indicated a significant difference. For this study, CUSUM graphs were generated from the three phases of the console time and the total time. The LC stages were determined from the times on the CUSUM graphs. All statistical calculations were performed with JMP statistical software, v. 13 (SAS Institute, Japan).

## 3 RESULTS

### 3.1 Patients' characteristics and perioperative outcomes

Thirty‐one consecutive patients (40 lesions) were analyzed. Table S1 shows the patients' characteristics, and Table S2 shows the patients' perioperative variables. There were no significant differences between the 20 lesions in the early period (15 patients) and the 20 lesions in the late period (16 patients). To reinforce the inguinal region, 26 lesions were treated with self‐fixating mesh and 14 lesions were fixed with polypropylene mesh. Eleven lesions were fixed with tacking and three with sutures. In the first 11 lesions, polypropylene mesh was fixed using a tacker that was familiar with L‐TAPP. Next, we changed to self‐fixing mesh. Along with the operator's proficiencies in the skill of R‐TAPP, the polypropylene mesh was fixed with sutures.

### 3.2 Evaluating the learning curve using CUSUM

**FIGURE 5:**
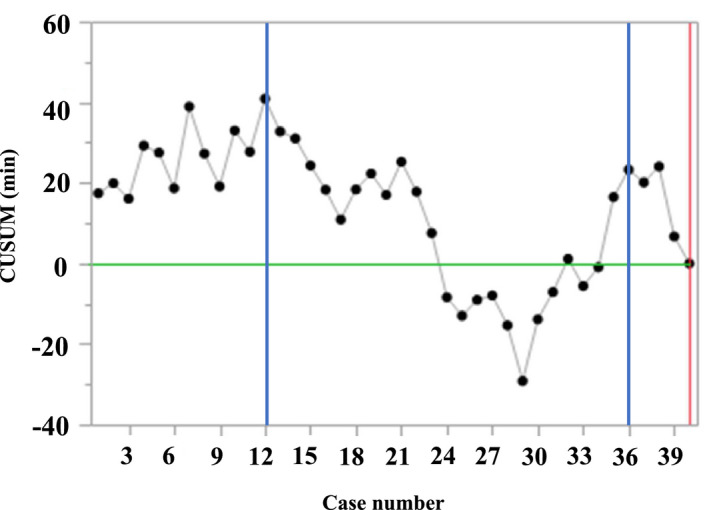
The CUSUM graph of the total time for phase‐identified changes in slope at lesions 12 and 36, which divided the learning curve into three distinct stages

The median time required for the peritoneal incision phase was 30 min (range, 18–54 min), that for the mesh placement phase (including fixation) was 13 min (range, 6–30 min), that for the peritoneal suturing phase was 10 min (range, 3–28 min), and that for total time for all phases was 54 min (range, 33–86 min; Table S2). The CUSUM graph for the total time of phase‐identified changes in slope at lesions 12 and 36 divided the LC into three distinct stages (Figure 5). In each phase, the CUSUM graph also identified changes in slope at lesions 10 and 35, which divided the LC into three distinct stages (Figure S1–S3).

## 4 DISCUSSION

The advantages of a bipolar dissection technique in R‐TAPP are the ability to maintain adequate operative field control with both hands, delicate dissection, and efficient hemostasis. As a result, we consider that peritoneal flap creation and parietalization of spermatic cord components may be performed more safely. R‐TAPP is usually performed with the left hand using fenestrated bipolar forceps for grasping and hemostasis, and with the right hand using a monopolar instrument (scissors/hook) for dissection (Intuitive Surgical). This method is difficult to perform because the surgical field is controlled only by grasping with the left hand. In addition, hemostasis with the left hand is inconvenient because of the poor surgical field, which leads to the potential of prolonged operative time owing to forceps change.

By evaluating the surgeon's LC after completing the LC for L‐TAPP, three different stages were observed. The phases of the LC and the total time changed at lesions 10 and 35. The three stages indicated the surgeon's comfort operating the robot, competency, and mastery, respectively, as the surgeon developed experience. The three stages generated corresponded to the learning, competency, and mastery stages delineated in previous studies of surgical LC.^11^, ^12^

Our evaluation of the LC of a simple surgical technique excluded the effects of differences, such as surgical team performance or docking the da Vinci robotic system, other than operative or console time. Reports of completed LC for R‐TAPP by expert surgeons in L‐TAPP varied from 25 to 139 cases, but the majority of studies reported 30–50 cases, similar to our results.^13^, ^14^, ^15^, ^16^, ^17^, ^18^ We also performed 50 cases of RG during the same period. The LC in our report may be skewed, as it does not take into account the proficiency gained from using the robotic system in RG interspersed with R‐TAPP procedures. However, considering the widespread use of the DVSS and other systems, we believe that our results are more realistic because surgeons adopting robotic surgery in their practices are likely to incorporate this approach in several types of procedures. LC for R‐TAPP for surgeons performing robotic surgery for other diseases within the study period have been reported.^13^, ^14^ For example, Aghayeva et al^13^ reported that performing robotic surgery for other diseases may lead to a crossover effect that shortens the operation time for R‐TAPP.

The benefit of starting the flap creation in the cranial position is that it minimizes exposing the mesh to the abdominal contents. With other approaches, any hole in, or failure of, the peritoneal flap is located directly over the mesh, exposing the mesh to the bowel. This approach was possible with the enhanced endo‐wrist dexterity of the DVSS, and postoperative pain prevention was expected owing to the tackless mesh fixation.^19^

The heterogeneity of the cases potentially confounded our results, as each case involved a unique set of steps and mesh choice, and required certain skill sets, such as mesh fixation. However, considering the fact that hernia defects and adhesions differ, modifying the procedure while standardizing the technique is acceptable. There was no significant difference in outcomes between the early and late periods in this study. In addition, although two postoperative complications were observed in the late period, these were Clavien–Dindo classification grade 1.^20^

R‐TAPP using bipolar forceps by a surgeon who completed the LC for L‐TAPP had three performance stages and was feasible by lesion 35.

The limitations of this study are that it involved a retrospective cohort, a single surgeon's experience, and a small sample size. Further validation studies involving large sample sizes for various surgeons are needed.

## DISCLOSURE

Ethics Approval: The Aichi Medical University Ethics Committee and Medical Safety Management Office approved this study (Approval No. 2019‐086). This study was performed in accordance with the 1964 Declaration of Helsinki and its later amendments or comparable ethical standards. All patients provided signed informed consent before surgery.

Conflict of Interest: The authors declare no conflicts of interest for this article.

Author Contributions: Saito designed the study and wrote the initial draft of the article. Sano contributed to data interpretation and critical revision of the article for important intellectual content. All other authors (Y.F., S.K., and K.K.) contributed to the data collection and interpretation and critically reviewed the article. All authors have read and approved the final version of the article and have agreed to be accountable for all aspects of the study, ensuring that any questions related to the accuracy or integrity of any part of the work are resolved.

## Supporting information

Figure S1‐S3

Table S1‐S2

Video S1

Video S2

Video S3
