# Supplementary material for: How to Establish the Bipolar Forceps Dissection Method in Robotic Inguinal Hernia Repair
Source: Ann Gastroenterol Surg. 2021 Dec 14;6(3):454–9. doi: 10.1002/ags3.12535 (PMC9130915; doi:10.1002/ags3.12535)
Supplement: Supplementary file 1 — Figure S1‐S3 [file AGS3-6-454-s003.pptx]

## Slide 1
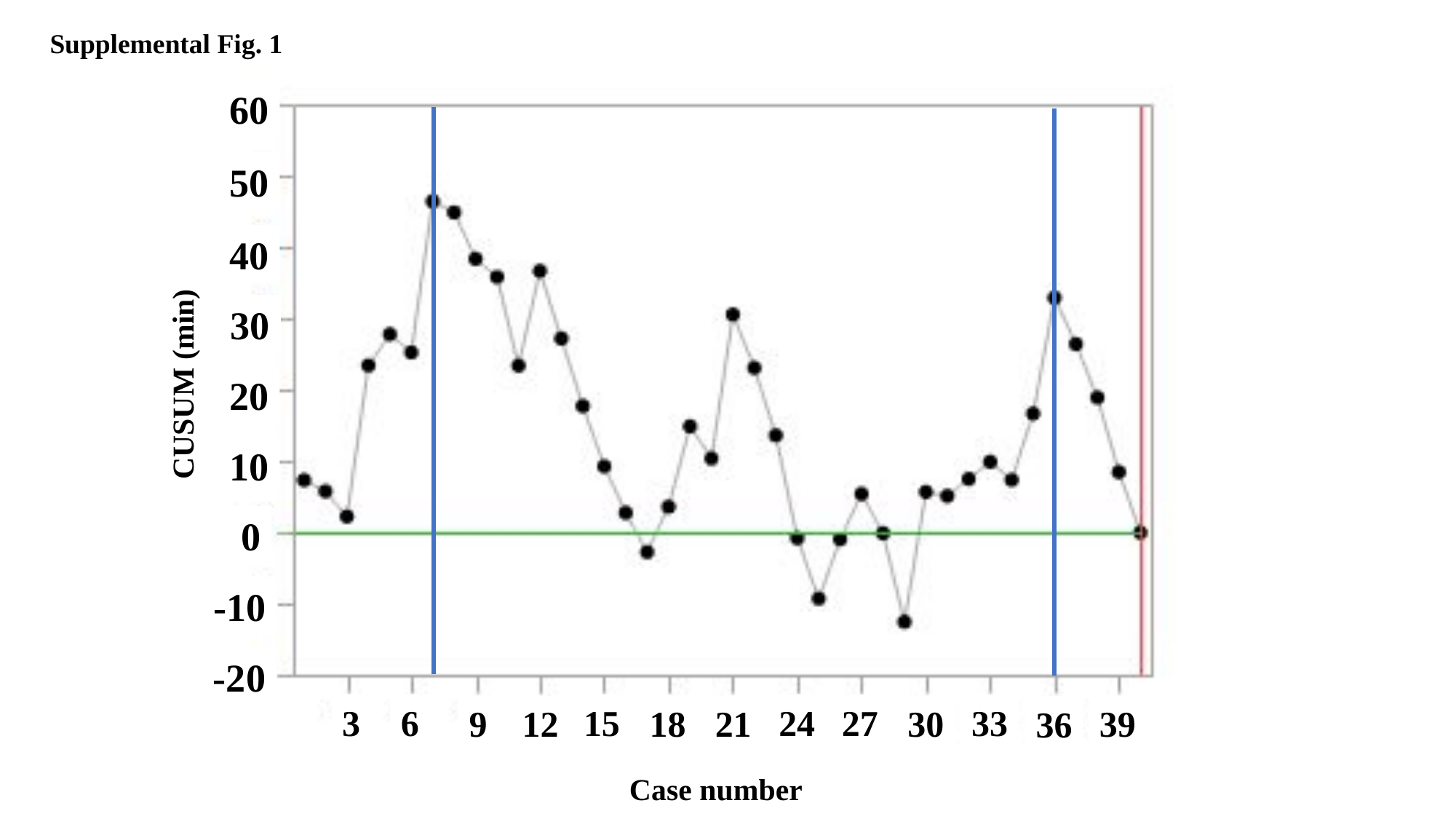

Supplemental Fig. 1
60
50
40
30
CUSUM (min)
20
10
0
-10
-20
3
6
15
24
27
33
9
12
21
39
18
30
36
Case number

## Slide 2
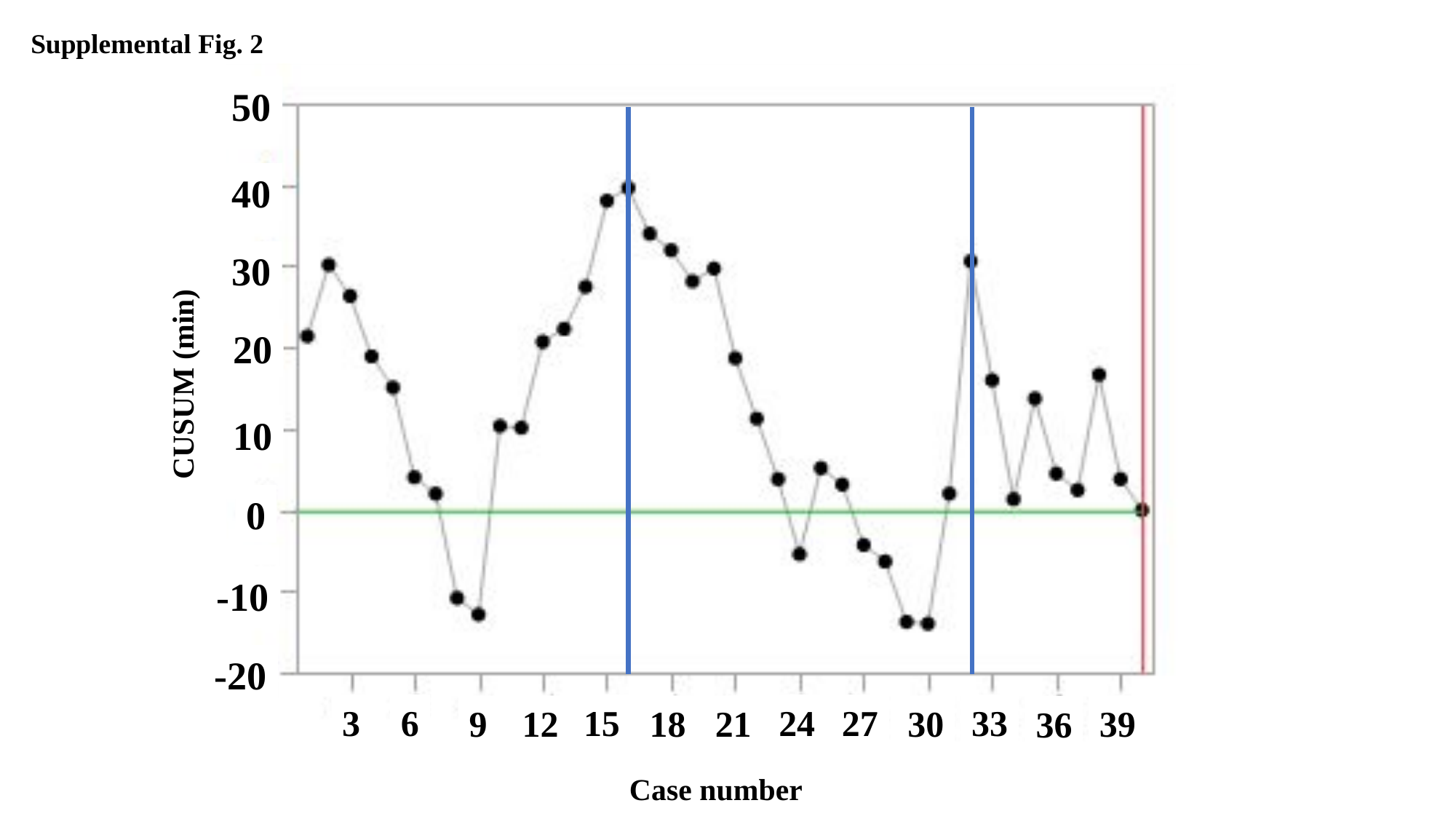

Supplemental Fig. 2
50
40
30
20
CUSUM (min)
10
0
-10
-20
3
6
15
24
27
33
9
12
21
39
18
30
36
Case number

## Slide 3
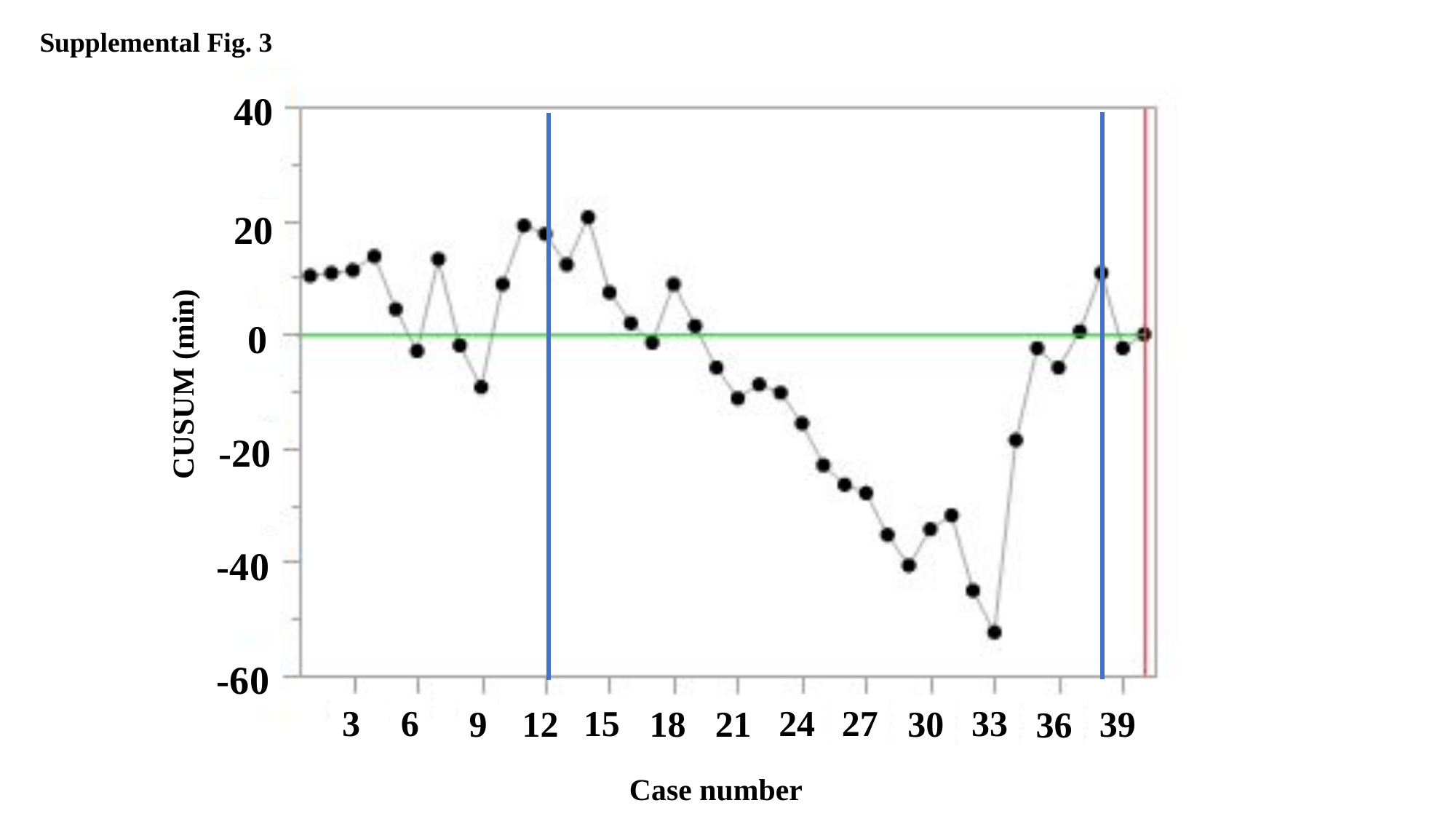

Supplemental Fig. 3
40
20
0
CUSUM (min)
-20
-40
-60
3
6
15
24
27
33
9
12
21
39
18
30
36
Case number
