# Supplementary material for: How to Establish the Bipolar Forceps Dissection Method in Robotic Inguinal Hernia Repair
Source: Ann Gastroenterol Surg. 2021 Dec 14;6(3):454–9. doi: 10.1002/ags3.12535 (PMC9130915; doi:10.1002/ags3.12535)
Supplement: Supplementary file 2 — Table S1‐S2 [file AGS3-6-454-s001.pptx]

## Slide 1
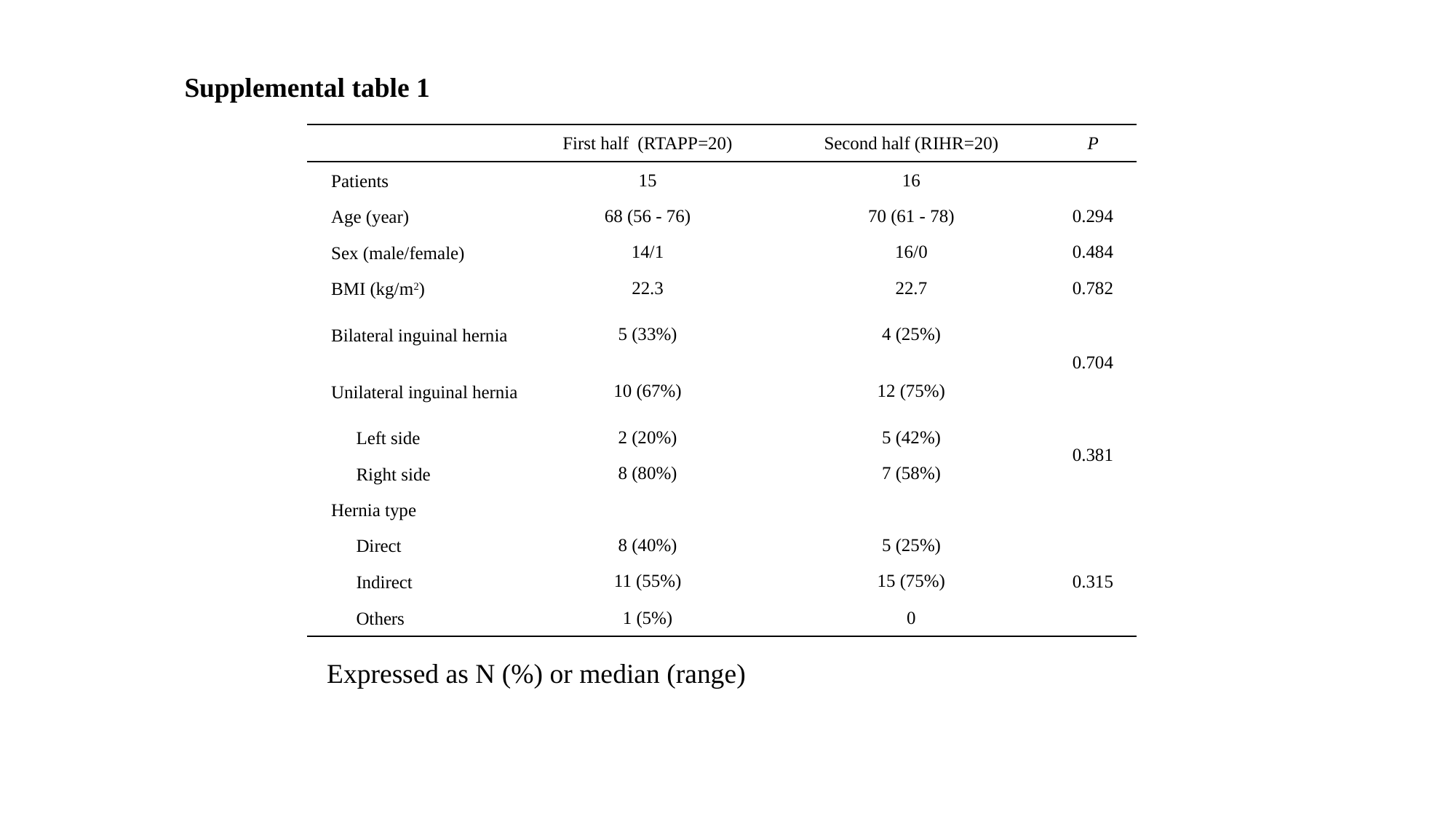

Supplemental table 1
| | | First half (RTAPP=20) | Second half (RIHR=20) | P |
| --- | --- | --- | --- | --- |
| Patients | | 15 | 16 | |
| Age (year) | | 68 (56 - 76) | 70 (61 - 78) | 0.294 |
| Sex (male/female) | | 14/1 | 16/0 | 0.484 |
| BMI (kg/m2) | | 22.3 | 22.7 | 0.782 |
| Bilateral inguinal hernia | | 5 (33%) | 4 (25%) | 0.704 |
| Unilateral inguinal hernia | | 10 (67%) | 12 (75%) | |
| | Left side | 2 (20%) | 5 (42%) | 0.381 |
| | Right side | 8 (80%) | 7 (58%) | |
| Hernia type | | | | |
| | Direct | 8 (40%) | 5 (25%) | 0.315 |
| | Indirect | 11 (55%) | 15 (75%) | |
| | Others | 1 (5%) | 0 | |
Expressed as N (%) or median (range)

## Slide 2
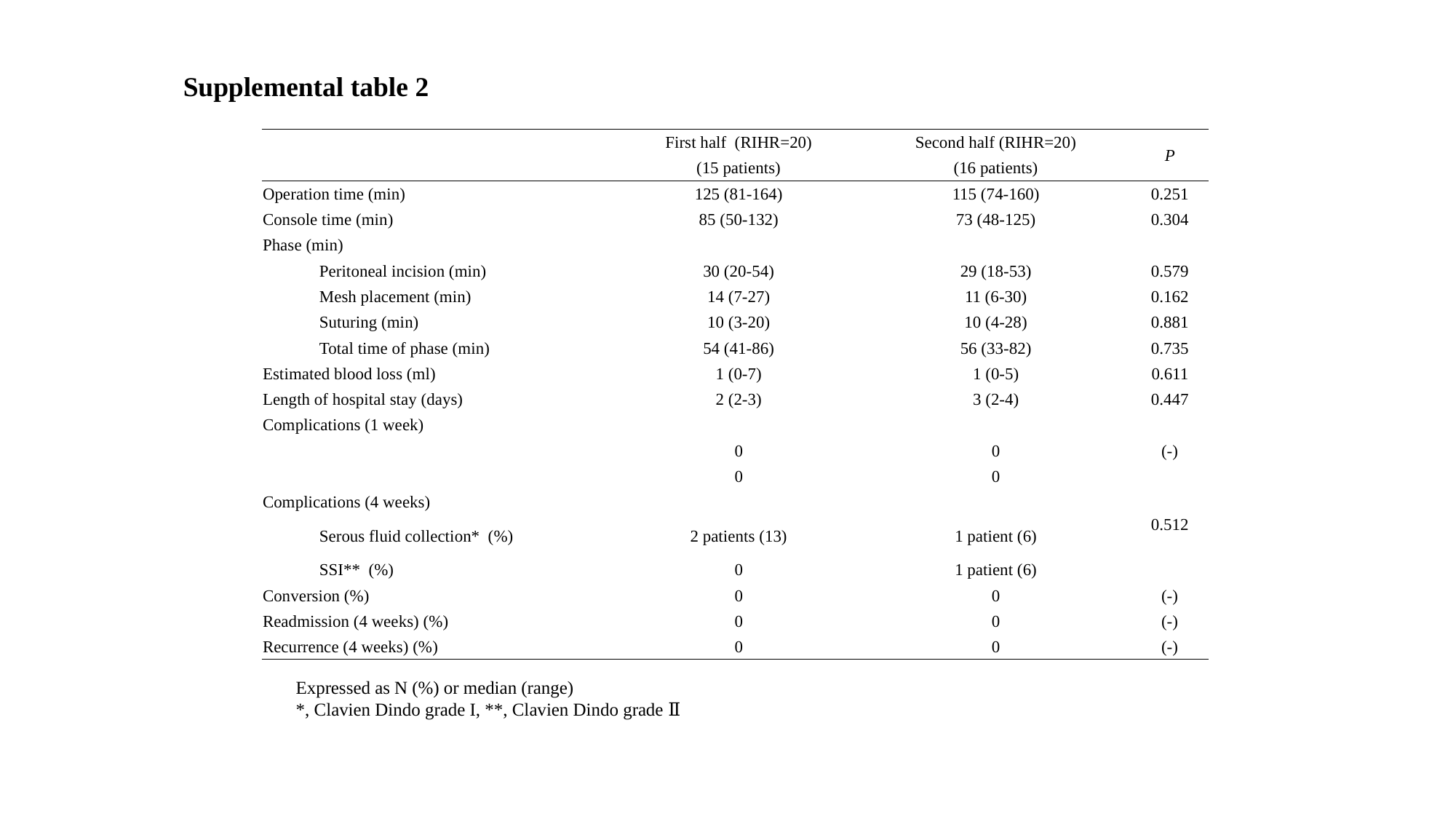

Supplemental table 2
| | | First half (RIHR=20) | Second half (RIHR=20) | P |
| --- | --- | --- | --- | --- |
| | | (15 patients) | (16 patients) | |
| Operation time (min) | | 125 (81-164) | 115 (74-160) | 0.251 |
| Console time (min) | | 85 (50-132) | 73 (48-125) | 0.304 |
| Phase (min) | | | | |
| | Peritoneal incision (min) | 30 (20-54) | 29 (18-53) | 0.579 |
| | Mesh placement (min) | 14 (7-27) | 11 (6-30) | 0.162 |
| | Suturing (min) | 10 (3-20) | 10 (4-28) | 0.881 |
| | Total time of phase (min) | 54 (41-86) | 56 (33-82) | 0.735 |
| Estimated blood loss (ml) | | 1 (0-7) | 1 (0-5) | 0.611 |
| Length of hospital stay (days) | | 2 (2-3) | 3 (2-4) | 0.447 |
| Complications (1 week) | | | | |
| | | 0 | 0 | (-) |
| | | 0 | 0 | |
| Complications (4 weeks) | | | | |
| | Serous fluid collection\* (%) | 2 patients (13) | 1 patient (6) | 0.512 |
| | SSI\*\* (%) | 0 | 1 patient (6) | |
| Conversion (%) | | 0 | 0 | (-) |
| Readmission (4 weeks) (%) | | 0 | 0 | (-) |
| Recurrence (4 weeks) (%) | | 0 | 0 | (-) |
Expressed as N (%) or median (range)
*, Clavien Dindo grade I, **, Clavien Dindo grade Ⅱ
